# Revalorization of sunflower stalk pith as feedstock for the coproduction of pectin and glucose using a two-step dilute acid pretreatment process

**DOI:** 10.1186/s13068-021-02045-2

**Published:** 2021-10-01

**Authors:** Qibo Zhang, Lu Cheng, Xutong Ma, Xin Zhou, Yong Xu

**Affiliations:** 1 Jiangsu Co-Innovation Center of Efficient Processing and Utilization of Forest Products, Nanjing Forestry University, Nanjing, 210037 People’s Republic of China; 2 College of Chemical Engineering, Nanjing Forestry University, No. 159 Longpan Road, Nanjing, 210037 People’s Republic of China; 3 Jiangsu Province Key Laboratory of Green Biomass-Based Fuels and Chemicals, Nanjing, 210037 People’s Republic of China

**Keywords:** Sunflower stalk pith, Two-step acid treatment, Pectin, Glucose, Enzymatic hydrolysis

## Abstract

**Background:**

Sunflower stalk pith, residue from the processing of sunflower, is rich in pectin and cellulose, thereby acting as an economic raw material for the acquisition of these compounds. In order to increase the commercial value of sunflower processing industry, a two-step dilute sulfuric acid treatment process was conducted on spent sunflower stalk pith to obtain the value-added products, pectin and glucose.

**Results:**

In this study, pectin was firstly extracted under mild acid condition to avoid pectin degradation, which was conducted at 90 °C with a pH of 2.0 for 2 h, and ~0.14 g/g of pectin could be recovered. Then the remaining solids after pectin extraction were subjected to the reinforced treatment process with 0.75% H_2_SO_4_ at 150 °C for 30 min to further improve enzymatic hydrolysis efficiency. Moreover, by combining a fed-batch enzymatic hydrolysis strategy, a solid loading content of 16% was successfully achieved and the glucose titer reached 103.1 g/L with a yield of 83.6%.

**Conclusion:**

Finally, ~140 g pectin and 260 g glucose were produced from 1 kg of raw sunflower stalk pith using the integrated biorefinery process. This work puts forward a two-step dilute acid pretreatment combined with enzymatic hydrolysis method to produce pectin and glucose from sunflower spent waste.

**Supplementary Information:**

The online version contains supplementary material available at 10.1186/s13068-021-02045-2.

## Background

The food processing industry generates abundant waste, which as renewable precursor sources, are attracting attention for their implementation in the development of production of green materials, fuels, and chemicals [1]. These environmentally friendly resources are gaining traction as a complementary energy feedstock to avoid competition with the crops grown for human food or fuels [2, 3]. Sunflower, as the third largest source in worldwide vegetable oil, is extensively cultivated [4]. Correspondingly, a large amount of lignocellulosic residues, including sunflower heads, leaves and stalks, were generated; however, sunflower processing residues are poorly utilized and even burned directly in the fields, which cause a waste of biomass resources and an increase in environmental pollution [5, 6]. Thus, a deep-processing of sunflower processing residues towards to high-valued products is required for sustainable development goals.

Sunflower stalk pith (SSP), as the primary byproducts of sunflower processing industry, are composed of a high content of pectin (15–20%) and cellulose (35–45%) and a low content of hemicellulose (5–10%) and lignin (3–5%) [7, 8]. Herein, pectin is a structural hetero-polysaccharide contained in the primary cell walls of terrestrial plants, mostly vegetables and fruits [9]. Pectin is well known as stabilizers in food industries due to its ability to gel and give viscosity, and the use of pectin in food formulations or pharmaceuticals industry has beneficial effects on human health such as stimulating the immune response, reduces the cholesterol absorption in the blood and lower cancer risk [10, 11]. These benefits lead to a high price that exceeded $21/kg, justifying the search for new raw pectin-extraction materials; therefore, the extraction of high value-added pectin products from SSP can potentially increase the commercial value of sunflower processing industry [10]. In general, the commercial pectin is recovered using acidic hot water with mineral acids, such as HCl and H_2_SO_4_, at a mild temperature range of 60–90 °C with a low pH condition [12, 13]. After the pectin has been extracted, a large amount of cellulose (a polymer composed of repeating units of glucose) is still retained in the remaining solids, and it can be hydrolyzed enzymatically to glucose, which is easily transformed to the other value-added biochemicals [14].

However, the processing of mildly acidic extraction of pectin cannot render cellulose accessible by subsequent enzymatic activities. It is also essential to develop an integrative and efficient process to disrupt the lignocellulosic matrix of biomass and render cellulose more accessible to further enzymatic attack [15]. Among various pretreatments, dilute acid hydrolysis is the most frequently studied process for biomass, and it has been regarded as a suitable and the most feasible technology for bioethanol production on an industrial scale [16]. Therefore, considering it comprehensively, a two-step biorefinery concept was proposed via multiple product formation by (i) a slightly acidic extraction process using dilute H_2_SO_4_ as a solvent to break the SPP cell walls to release pectin by offering disruptive shear forces; and (ii) a reinforced acidic treatment process for pectin-extracted solids using dilute H_2_SO_4_, which can boost the enzymatic hydrolysis efficiency (Additional file 1: S1).

## Results and discussion

### Pectin extraction from sunflower pith with acid, alkali and enzyme

**Fig. 1:**
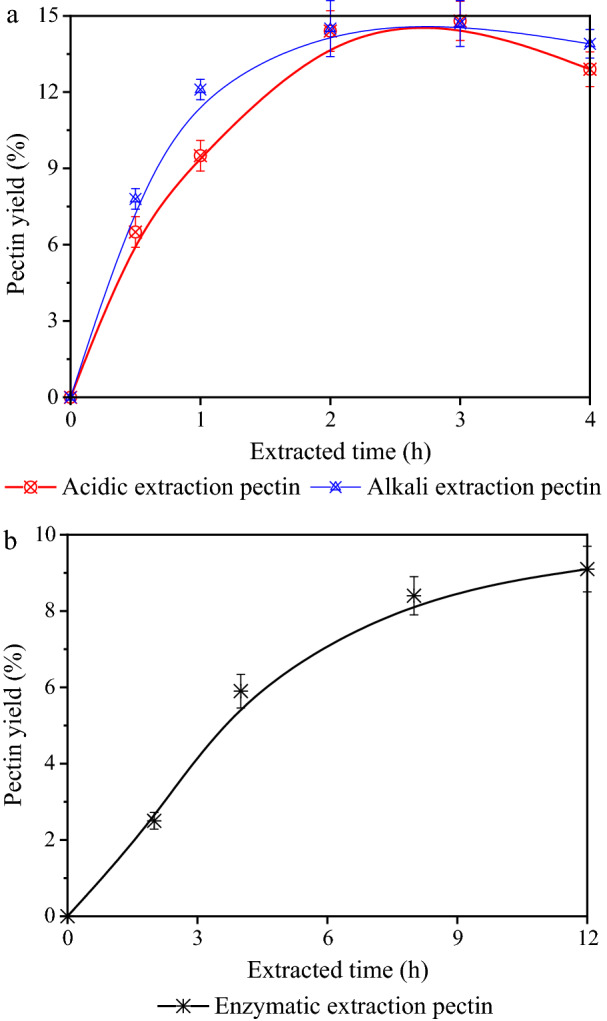
Yields of pectin from SSP with different extraction strategies. **a** Acidic and alkali extraction; **b** enzymatic extraction. Standard errors calculated from triplicates

In order to squeeze the maximum quantity of pectin products from sunflower pith materials, acidic- (pH 2.0, 90 °C and 0.5–4.0 h), alkaline- (pH 10.0, 90 °C and 0.5–4.0 h) and enzymatic- (cellulase, pH 4.5, 50 °C and 2–12 h) extraction strategies were first compared in this study [17, 18]. The results from different extraction strategies are shown in Fig. 1a, b (Additional file 1: S2). They suggested that these methods could be used to extract pectin with a wide range of yield of 2.5–14.8%, and the maximum yield from acidic extraction is higher than that of alkaline and enzymatic extraction. It could be observed that the efficiency of pectin extraction by cellulase was lower than acid and alkali. Apparently, the extraction time was not satisfied to adequately release pectin, which resulted in a relatively low yield. Therefore, enzymatic extraction strategy is not suitable for industrial-scale pectin production because of the high costs of enzyme and time. Although the maximum pectin yields from acidic extraction and alkaline extraction were similar, it could be found that the average molecular weight (*Mw*) of alkali-extracted pectin sample (21.2 kDa, extraction time 2 h) was significantly lower than that of the acid-extracted pectin (33.2 kDa, extraction time 2 h) and cellulase-extracted pectin (31.5 kDa, extraction time 12 h). Since the pectin chain is degraded easily through a β-elimination reaction in the case of the alkaline condition, the Mw of alkali-extracted pectin was lower [19]*.* Generally, the viscosity of pectin was positively correlated with the Mw and the high Mw pectin is more conducive to emulsifying activities. Overall, acidic extraction strategy is more suitable for extracting pectin from SSP materials and the sulfuric acid, as the most commonly used inorganic acid, owing to its low cost, was used for pectin extraction in this study.

Figure 1a indicates that the yield of acid-extracted pectin increased linearly with the increase in duration of extraction over the range of 0.5–2.0 h, and the yield of pectin did not consistently increase while the extraction time more than 2 h, and it even decreased when the duration of extraction was excessive. A similar phenomenon was obtained by Yapo et al. that the overlong extraction time for pectin from sugar beet resulted in large molecules were almost completely degraded into smaller sized ones [20]. It was deduced that the pectin obtained in the extraction medium had been destroyed and disintegrated [21]. Overall, the extraction time was fixed at 2 h for the pectin extraction experiments to minimize the cost, which a yield (g pectin/g substrate) of 0.143 could be obtained.

**Fig. 2:**
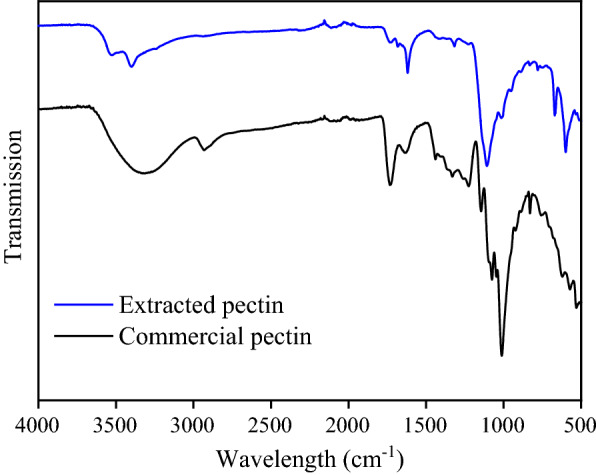
FTIR spectra of sunflower pith pectin and commercial pectin

Since the presence of high content galacturonic acids, therefore, pectic materials all have a characteristic fingerprint region in Fourier transform infrared (FTIR) spectroscopy [22], the distinctive peaks shown for galacturonic acids in Fig. 2 are at 969 cm^−1^ (C–O-bending), 1020 cm^−1^ (C–O–H deformation), which confirmed that its identity was pectin [7, 18]. The FTIR spectrum of extracted pectin sample from SSP was compared with commercial pectin and the results shown in Fig. 2. The shape of spectrum result for extracted pectin from SSP was quite similar to that of commercial pectin sample. Moreover, the HPLC analysis showed that the galacturonic acid content was ~67% from the SSP. All evidence confirmed that extracted substance is the pectic polysaccharide. Although the average *Mw* of SSP-pectin (33.2 kDa) was lower than that the values of commercial pectin from apple or citrus, it was still consistent with previous studies that show that the average *Mw* of pectic materials from various sources, which typically in the range of 10–100 kDa, and can be used as value-added food additives [23].

### Reinforced acid pretreatment for improving the efficiency of enzymatic hydrolysis

Acidic solution with pH 2.0 at a concentration of 0.005 mol/L H_2_SO_4_ was employed to extract pectin, therefore, the process of pectin extraction also could be regarded as a slightly acidic prehydrolysis for SSP. After the slightly acidic prehydrolysis, approximately 95.8% of the glucan was retained in SSP residues after pectin extraction, and the relative content increased from 32.3% to 51.5%. The retained glucan in pretreated solids can be enzymatically hydrolyzed into glucose; subsequently, glucose is able to be bioconverted into ethanol or other biochemicals. It is well known that pectin is the major component of the primary cell walls, and the main function of pectin is hydration and adhesion of wall cell. Thus, the presence of pectin can influence the porosity of cell wall and morphogenesis of plant. Pectin, as a physical barrier, restrict the access of enzymes to the cellulosic part of cell wall to a certain extent [24, 25]. Namely, pectin can affect the accessibility of cellulases. Thus, to verify that the removal of pectin improves the saccharification of cellulose, the raw SSP and solids after pectin-extracted SSP (PE-SSP) were all offered to enzymatic hydrolysis process with a loading dosage of 4% (w/v).

**Fig. 3:**
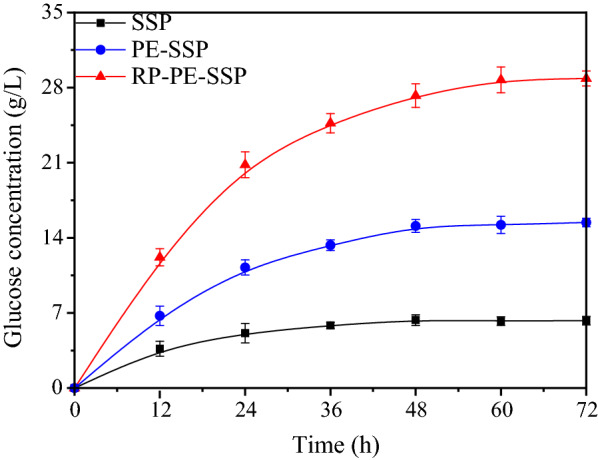
Time course of enzymatic hydrolysis with 4% solids dosage of SSP, PE-SSP and RP-PE-SSP. Standard errors calculated from triplicates

It can be observed that in Fig. 3, 6.28 g/L glucose was accumulated with an enzymatic hydrolysis yield of 44.2% at 72 h, indicating that the raw SSP was poorly digested by the enzymes. Correspondingly, a glucose yield of 68.1% was obtained from enzymatic hydrolysis of the 4% PE-SSP solids. Apparently, the slightly acidic prehydrolysis process with 0.005 mol/L H_2_SO_4_ could improve the enzymatic hydrolysis efficiency with the remove of pectin. While the pectin extraction strategy improved the yield of enzymatic hydrolysis, the results have not been entirely satisfactory for generating the maximum quantity of profit-generating products.

**Fig. 4:**
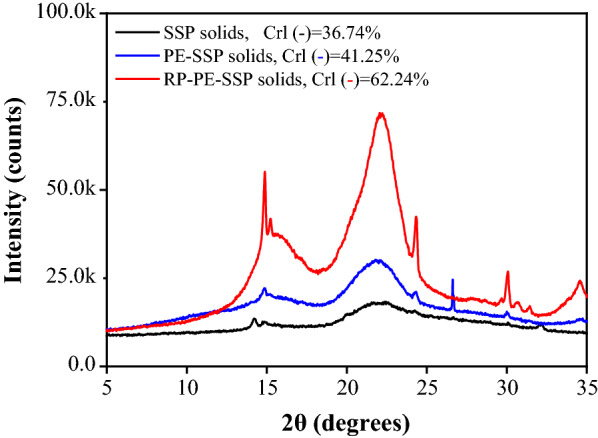
XRD patterns obtained from the SSP, PE-SSP and RP-PE-SSP

The crystallinity of cellulose is known to significantly affect the digestibility of cellulases. The XRD diffraction analysis, as shown in Fig. 4, showed the change in cellulose crystallinity of the SSP after acidic prehydrolysis. The diffraction intensity at 2*θ* = 22.1° represents the cellulose crystalline portion. Although, the diffraction intensity of SSP at 2*θ* = 22.1° increased after pectin extraction, the *CrI* of the PE-SSP solids sample (46.3%) increased merely 10% compared with that of the raw SSP material (36.7%). The results suggested that the PE-SSP required additional pretreatment to increase the *CrI* and improve the enzymatic accessibility for cellulose. Dilute acid pretreatment is the most frequently studied process for agricultural biomass and has been considered to be a suitable technology for bioethanol production at an industrial scale [15]. Thus, a complementary step, aimed at improving the efficiency of enzymatic hydrolysis, was performed with 0.75% (w/v) H_2_SO_4_ at 150 °C for 30 min [4].

**Fig. 5:**
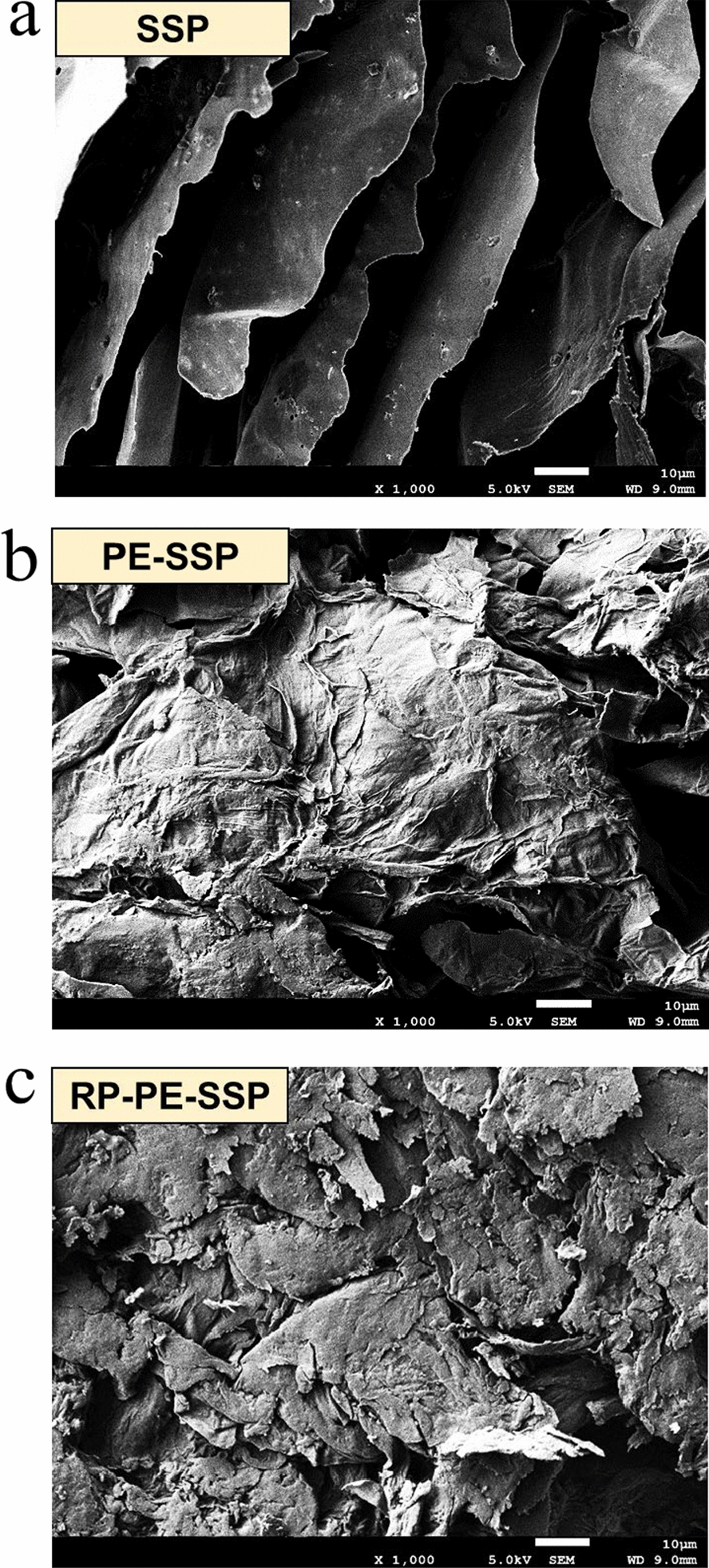
Scanning electron micrographs of **a** SSP, **b** PE-SSP, and **c** RP-PE-SSP solids

The composition analysis of the reinforced pretreated PE-SSP (RP-PE-SSP) solids showed that the relative content of glucan increased significantly from 51.5% to 71.1% with a recovery yield of 94.1%, and the xylan content decreased from 7.2% to 3.1%. Moreover, the peak of reinforced pretreatment solids was sharper, and the *CrI* increased substantially to 62.4%. In addition, the physical structure of the SSP raw material, PE-SSP and RP-PE-SSP residues were studied by using scanning electron microscope (SEM). As shown in Fig. 5a, the surface morphologies of SSP were smooth and highly ordered. Observation of Fig. 5b showed that the surface morphologies of PE-SSP changed slightly, and only a small part of the cellulose was exposed, while RP-PE-SSP (Fig. 5c) depicted a highly unstructured rough surface with a substantial amount of cracks.

Large specific surface area and total pore volume can offer high adsorption capacity for cellulases, therefore, the specific surface area and pore volume of lignocellulosic materials are also considered to be the key factors impacting the enzymatic hydrolysis. The specific surface area of raw SSP, PE-SSP and RP-PE-SSP were 1.529, 2.803, and 3.591 m^2^/g, respectively, and the corresponding total pore volume was 0.0026, 0.0051, and 0.0064 cm^3^/g, respectively. The result depicted that the specific surface area and total pore volume significantly increased by enhancement of pretreatment intensity. Such an increase can be attributed to the partial breakdown of the raw material microstructure, resulting in the formation of more cracks and larger pore size, as evident from the micrographs of SEM [26]. Enzymatic hydrolysis is known to be strongly affected by porosity, including specific surface area and total pore volume, since the cellulase can directly contact the cellulose structure through the pores. Overall, incremented specific surface area and pore volume percentage allow better access of the cellulase to inner portion of lignocellulosic solids. Visual observations show that reinforced pretreatment efficiently dissected the physical structure after reinforced pretreatment with 0.75% H_2_SO_4_ at 150 °C for 0.5 h, which resulted in an enzymatic hydrolysis yield of up to 92.3% at 72 h with a loading dosage of 4% RP-PE-SSP solids. Obviously, reinforced pretreated can enhance interfacial interactions between the solids and cellulases, resulting in a significantly improvement for enzymatic hydrolysis efficiency [27, 28].

### Enzymatic hydrolysis with batch and fed-batch modes

The fermentable sugars should be at levels as high as possible in the industrial-scale utilization of lignocellulosic materials due to high final sugars is beneficial in improving the utilization rate of equipment, and lowers the consumption of water and energy. Using bioethanol generation as an example, obtaining fermentation broths of at least 4% (w/v) of ethanol is essential for economical large-scale production owing to the costs of ethanol purification, which dramatically increase when the ethanol titer is lower [29]. Thus, in this biorefinery framework, at least more than 80 g/L of total reducing sugars obtained from the biomass is required. Correspondingly, enzymatic hydrolysis must be conducted with a pretreated solid loading of more than 10–20% (w/v) on the basis of the cellulose proportion. Moreover, high-solids enzymatic hydrolysis is preferred for economic reasons for biorefinery processes on an industrial scale. Thus, enzymatic hydrolysis assays with batch mode were first performed with 4, 8, 12, and 16% RP-PE-SSP solids loading to produce a high titer of glucose.

**Fig. 6:**
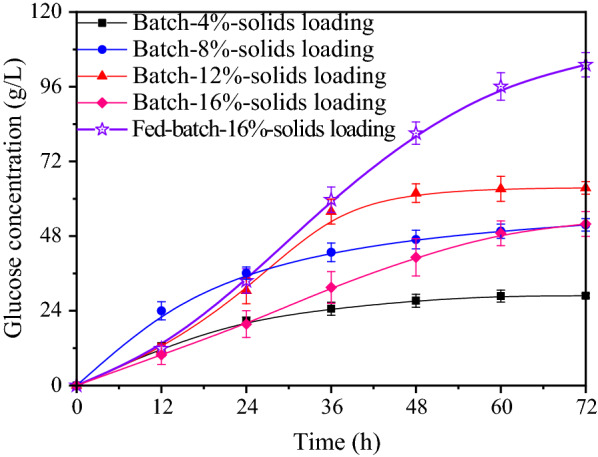
Time course of batch enzymatic variety at different solid loadings (4–16%) and a fed-batch enzymatic hydrolysis with total 16% solids dosage of RP-PE-SSP. Standard errors calculated from triplicates

Figure 6 shows that a glucose titer of 28.9, 51.6, 63.5, and 54.4 g/L final glucose accumulated with batch operation at the solid loading of 4, 8, 12, and 16%, respectively. In the enzymatic hydrolysis process, it was observed that 4% solid in the system could be liquefied within 8 h. However, the mixing became fouled with a batch operation of more than 8% (w/v) solids loading, and in the case of 12% (w/v) RP-PE-SSP solids loading, the reaction medium was not mixed at the beginning of the experiments, even with a significant increase in the rate of agitation. In addition, the system with high solids loading was difficult to liquefy even when treated for more than 24 h, which is speculated to be due to the reduction of crystallinity and the limited catalytic sites for cellulases [30, 31]. It is apparent that a high consistency of hydrolysis may result in difficulties of mass transfer owing to high viscosity as a result of high solids loading and the lack of free water in the enzymatic system. Especially for batch operation with 16% solids loading, over 10% solids (dry weight) were still remained in the enzymatic hydrolysis system and hydrolysate was very difficult to be collected due to the water absorption of the cellulose. From the summarized data, the enzymatic hydrolysis yields from the loading of 4, 8, 12, and 16% solids with batch operation were 92.2, 82.5, 67.7 and 43.4%, respectively. Apparently, high initial solids loading resulted a linear decline.

Although increasing the solids loading was the simplest and most direct way to enable a high concentration of sugars, this technique resulted in high viscosity, poor mixing, heat transfer and enzyme distribution problems, which reduced the efficiency of enzymatic hydrolysis [30, 32]. Correlatively, the fed-batch operation is regarded as an effective way to minimize these negative effects. Thus, we conducted a fed-batch enzymatic hydrolysis of RP-PE-SSP material to produce glucose. A fed-batch operation with a total of 16% solids dosage (4% every 12 h) was conducted for 72 h for enzymatic hydrolysis. When the enzymatic hydrolysis was successfully performed with a final solids content as high as 16% (w/v), the glucose titer obtained and yield reached 103.1 g/L and 83.6%, respectively. Overall, the fed-batch strategy enables an easy dynamical load and the production of a solution with a high glucose titer. The mass balance of the whole process for releasing pectin and glucose showed that a total of 140 g pectin and 260 g glucose were recovered from 1000 g raw material (dry weight) using a two-step pretreatment technology and the fed-batch enzymatic hydrolysis strategy.

## Conclusions

The extraction of pectin and glucose from SSP using a two-step dilute acid pretreatment combined with enzymatic hydrolysis was presented. As a first step, slightly acidic prehydrolysis is employed for extracting pectin; subsequently, in order to deconstruct the tight and complex structure of the residue from the first step, a reinforced pretreatment method with dilute acid was also be introduced and the fed-batch enzymatic hydrolysis achieved a high titer glucose accumulation. The entirety of the results clearly suggests that the two-step acid treatment with H_2_SO_4_ is a profitable option for the further exploitation of sunflower residue.

## Materials and methods

### Materials

The raw and dry SSP was ground to a particle size of 60–100 meshes. The chemical composition of SSP by percentage of weight was: 32.25% cellulose, 10.36% hemicellulose, 2.87% lignin, and 19.31% ash, and composition content all were based on an oven dry weight basis.

### Pectin extraction assays

First, 4 g SSP powder (dry weight) was mixed with 120 mL deionized water at a solid-to-liquid ratio of 1:30 (w/v) in a 1-L beaker while magnetically stirred. For acidic and alkali extraction, H_2_SO_4_ and NaOH were added to adjust the pH to 2.0 and 10.0, respectively; then the assays were performed in a 90 °C water bath for 0.5–4.0 h while stirred with an agitator at 160 rpm. For enzymatic extraction, cellulase (powder, 5000 U/g solid, Sigma, Shanghai, China) with a dosage of 150 U/g SSP powder was added in the system and the assays were conducted at pH 4.8 and 50 °C for 2–12 h. After pectin extraction process, the solid and liquid fractions were separated by vacuum filtration, the liquid fraction was used to collect pectin, and the solid fraction was dried and stored for further treatment. The liquid fraction was added with the same volume of absolute ethanol, and the pH was adjusted to 3.5 with KOH. This is the pH value at which pectin is minimally soluble. The pectin was precipitated after 12 h of sedimentation and then harvested by centrifugation at 6000×*g* for 15 min; the precipitated pectin was also washed with 70% (v/v) ethanol, centrifuged again (6000×*g* for 15 min at 4 °C), and dried at room temperature for 24 h [33].

### Reinforced dilute sulfuric acid pretreatment

The pectin-extracted SSP (PE-SSP) (3 g dry weight) was firstly mixed with 30 mL 0.75% (w/w) H_2_SO_4_ (solid/liquid: 1:10 [w/v]) in a 50 mL stainless steel vessel reactor. Then the sealed stainless reactor was immersed in glycerol bath and performed at 150 °C for 30 min. Once the pretreatment finished, the stainless steel vessel reactor was cooled to the room temperature by immersing in cold water bath. Finally, the solid after reinforced pretreatment of PE-SSP (RP-PE-SSP) and liquid fractions were separately collected by vacuum filtration (Additional file 1: Fig S1).

### Enzymatic hydrolysis assays

The enzymatic hydrolysis assays were all performed in a stirred reactor with a 0.5-L round-bottom flask warmed by a 50 °C hot plate and stirred for 72 h at 150 rpm using a two blade propeller. Three different substrates (solid fractions of raw SSP, PE-SSP and RP-PE-SSP) were subjected with 4% solids dosage. In addition, batch and fed-batch experiments were evaluated the RP-PE-SSP. Four batch enzymatic hydrolysis assays with different initial solids loading (4, 8, 12 and 16%) (dry basis) were directly added for enzymatic hydrolysis. Fed batch operation with a 4% (w/v) of initial solids (dry basis) content was first conducted, three loads of solids and cellulase were simultaneously fed with intervals of 12 h. The amount of insoluble solids and enzymes in each load was the same used in an equivalent batch process at 4% (w/v) solids and the final solids loadings was 16% [31]. The working volume for all the enzymatic hydrolysis experiments was 100 mL. 100 mL of 0.05 mol/L sodium citrate buffer was loaded to maintain the pH at 4.8 and 200 μL of 20 mg/mL sodium azide was also added to prevent microbial growth. The enzyme of cellulase (C2730, Celluclast^®^ 1.5 L, Novozymes, Sigma Co., Shanghai, China) was 20 FPIU/g-glucan. In addition, the solid residue was rinsed off with water until a neutral pH was attained and then subjected to enzymatic hydrolysis process.

### Analytical methods

The contents of cellulose, hemicellulose and total lignin for different materials were analyzed according to standard two-step acid hydrolysis method, which provided by the U.S. National Renewable Energy Laboratory (NREL) [34]. Briefly, 0.3 g of 20–80 meshes samples (SSP, PE-SSP and RP-PE-SSP) were pre-acid-hydrolyzed by 72% (w/w) H_2_SO_4_ at 30 °C for 1 h. Then, the slurry was diluted by deionized water to a concentration of 4% (w/w) H_2_SO_4_ and immediately subject to autoclave at 121 °C for 1 h. Finally, the autoclaved slurry was cooled and then used for determination of carbohydrates and lignin.

Carbohydrates (glucose, xylose and cellubiose) and galacturonic acid were determined by high-performance liquid chromatography (Agilent1260, USA) equipped with an Aminex Bio-Rad HPX-87H column with a fluent 0.6 mL/min of 5 mmol/L H_2_SO_4_ as mobile phase. The weight average molecular weight (*Mw*) of the extracted pectin were analyzed by gel permeation chromatography (GPC) (1260 Infinity, Agilent Technologies.) equipped with an Aminex Bio-Rad 42A column and the molecular weight (*Mw*) was estimated using a calibration curve of standard dextrans. All samples were centrifuged at 10,000 rpm for 5 min to obtain supernatants, which were then diluted and filtered through a 0.22-μm filter for HPLC and GPC analysis. The yields of pectin and glucose enzymatic hydrolysis were calculated as follows:

$$\text{Pectin yield} \, \left({\%}\right)\text{=}\frac{\text{Extracted pectin (g)}}{\text{Raw material (g)}} \times 100,$$

$$\text{Enzymatic hydrolysis yield}{ (\%)=}\frac{\text{Glucose content in enzymatic hydrolysate (g)}}{\text{Glucan in raw material (g)}} \times 100.$$

Fourier transform infrared (FTIR) spectra of the samples (Tensor 27-IR, Bruker, Billerica, MA, USA) was carried out using a Tensor 27-IR (Bruker, USA). 10 mg dry samples of extracted pectin, treated and untreated SPP solids were first tabled with 1 g KBr, and then the spectra of samples were scanned from 4000 to 500 cm^−1^.

Samples of the non-pretreated and pretreated SSP were sputter coated with gold/palladium using a SC7640 automatic high-resolution sputter coater and then subjected to scanning electron microscope (SEM) (FEI Quanta 400, Hitachi, Tokyo, Japan) for capturing different magnifications images [35].

The specific surface area and pore volume and pore size of the non-pretreated and pretreated SSP were determined using the Brunauer–Emmett–Teller (BET) based on nitrogen gas adsorption at 77 K [28]. In addition, all samples were dried using freeze dryer for 24 h and degassed under vacuum for 1 h before BET analysis,

The crystallinity of the non-pretreated and pretreated SSP were analyzed by using X-ray diffraction (XRD) with a range of 5°–35^o^ scattering angles (2*θ*) at a voltage of 40 kV and a current of 40 mA. And the followed equation was used to calculate the crystallinity index (*CrI*):

$$\text{CrI }\text{(\%) }= \frac{{I}_{002}-{I}_{am}}{{I}_{002}} \times 100,$$

in which ***I***_002_ refers to the maximum intensity of the crystalline cellulose I peak at 2*θ*≈22.5° and ***I***_am_ refers to diffraction intensity at amorphous portion evaluated as the minimum intensity between the main and secondary peaks [36].

The assays were all conducted in triplicate and the samples were measured in triplicate.

## Supplementary Information

**Additional file 1: Fig. S1** Graphic abstract: the scheme of the two-steps dilute acid pretreatment process for producing pectin and glucose**. Fig. S2 **The flowchart of pectin extraction by acid, alkali and cellulose.

## Data Availability

All data generated or analyzed during this study are included in this published article.

## Abbreviations

SSP: Sunflower stalk pith
PE-SSP: Pectin-extracted sunflower stalk pith
RP-PE-SSP: Reinforced pretreated pectin-extracted sunflower stalk pith
Mw: Molecular weight
BET: Brunauer–Emmett–Teller
FTIR: Fourier transform infrared
*CrI*: Crystallinity index
SEM: Scanning electron microscope
XRD: X-ray diffraction
HPLC: High-performance liquid chromatography
GPC: Gel permeation chromatography
