# Supplementary material for: Revalorization of sunflower stalk pith as feedstock for the coproduction of pectin and glucose using a two-step dilute acid pretreatment process
Source: Biotechnol Biofuels. 2021 Oct 1;14:194. doi: 10.1186/s13068-021-02045-2 (PMC8487133; doi:10.1186/s13068-021-02045-2)

**Additional file 1**

**Fig. S1** Graphic abstract: the scheme of the two-steps dilute acid pretreatment process for producing pectin and glucose


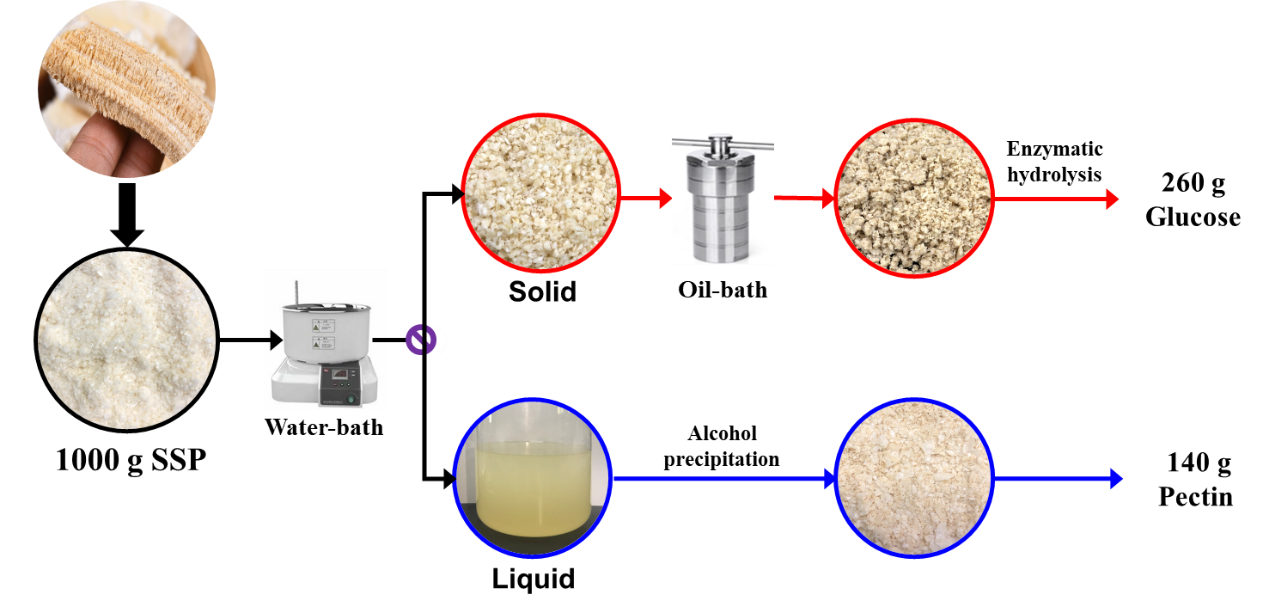


**Fig. S2** The flowchart of pectin extraction by acid, alkali and cellulase


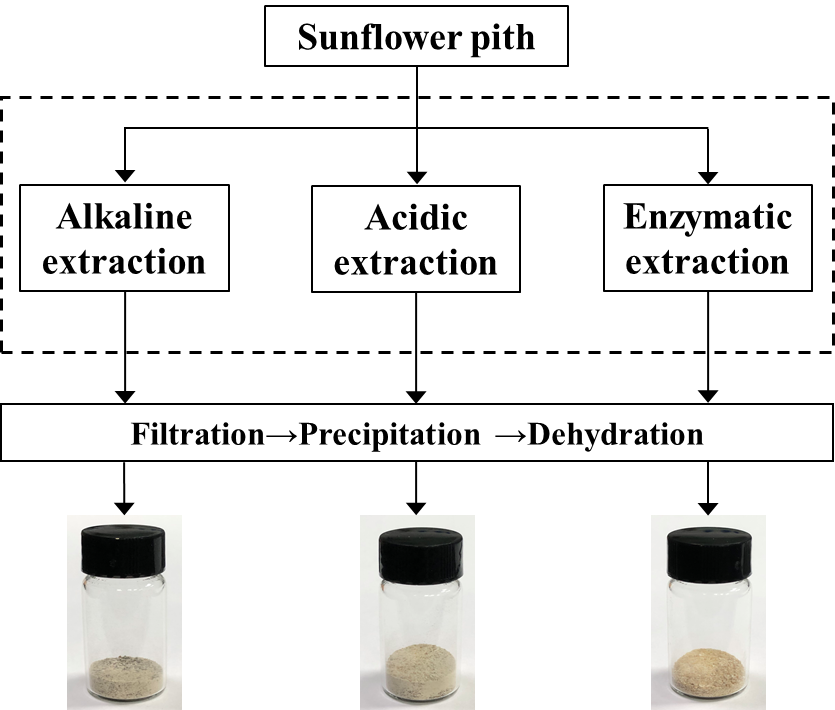

Supplement: Supplementary file 1 — Additional file 1: Fig. S1 Graphic abstract: the scheme of the two-steps dilute acid pretreatment process for producing pectin and glucose. Fig. S2 The flowchart of pectin extraction by acid, alkali and cellulose. [file 13068_2021_2045_MOESM1_ESM.docx]
